# Global Warming and the Spread of the Introduced Jellyfish *Cassiopea andromeda* : Thermal Niche and Habitat Suitability in the Mediterranean Sea

**DOI:** 10.1111/gcb.70548

**Published:** 2025-10-16

**Authors:** Lara M. Fumarola, Valentina Leoni, Guillaume Marchessaux, Gianluca Sarà, Stefano Piraino, Mar Bosch‐Belmar

**Affiliations:** ^1^ Consorzio Nazionale Interuniversitario Per Le Scienze Del Mare (Co.N.I.S.Ma.) Roma Italy; ^2^ Dipartimento Di Scienze e Tecnologie Biologiche Ed Ambientali (Di.S.Te.B.A), University of Salento Lecce Italy; ^3^ Aix Marseille Univ, Université De Toulon, CNRS, IRD Mio Marseille France; ^4^ Laboratory of Ecology, Department of Earth and Marine Science (DiSTeM), University of Palermo Palermo Italy; ^5^ National Biodiversity Future Center, Palermo (NBFC), 90133, University of Palermo Italy

**Keywords:** climate change, cnidaria, oxygen consumption, scyphistoma, thermal performance curve, upside‐down jellyfish

## Abstract

Climate change affects marine ecosystems in multiple ways, including sea warming and changes in biological community structure and diversity. The Mediterranean Sea has emerged as one of the most vulnerable regions, also because of the diverse patterns of introduction of non‐native species. First recorded in the coastal waters of Cyprus in 1903, the Red Sea jellyfish *Cassiopea andromeda* (Forskål, 1775) is spreading its distribution and local abundance, posing questions on its potential ecological implications. Here we identified the thermal tolerance, habitat suitability, and potential distribution range of the *C. andromeda* polyps, a key life cycle stage responsible for asexual reproduction and population persistence. By laboratory‐controlled respirometric measurements, we assessed that the polyps of *C. andromeda* exhibit their optimal metabolic performances at high water temperatures, but they are tolerant to winter conditions across the Mediterranean basin. Combining experimental respiration measurements with modelling approaches enabled the definition of the species' fundamental thermal niche, with an optimal seawater temperature at 35.7°C and critical limits at 6.4°C (minimum) and 39°C (maximum). Trait‐based thermal habitat suitability maps indicated a future increase of favourable habitats for the species under warming conditions according to the Representative Concentration Pathways (RCP 4.5 and 8.5 for 2050) in Mediterranean coastal areas. In the context of climate change scenarios, the rise of seawater temperature may enable polyps to thrive across a wider geographic range, predicting a westward and northward enlargement of *C. andromeda* populations in the Mediterranean Sea.

## Graphical abstract

Written Summary: Climate change is reshaping marine ecosystems, with the Mediterranean Sea among the most vulnerable due to warming and non‐native species such as the upside‐down jellyfish *Cassiopea andromeda* . We investigated the thermal tolerance and habitat suitability of its polyps, a key stage for reproduction and persistence. Respirometric experiments showed optimal performance at 35.7°C, with tolerance between 6.4°C and 39°C. Trait‐based habitat suitability maps, under present and future scenarios (RCP 4.5 and 8.5, 2050), indicated westward and northward enlargement of *C. andromeda* populations across the Mediterranean Sea.

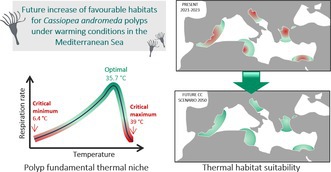

## 1 Introduction

Marine ecosystems are facing multiple impacts of climate change, including shifts in physicochemical properties (Lee et al. 2024) and wide biological effects, including organismal physiological stress responses, modified distributions of populations, and variations in community structure and organization (Doney et al. 2012; Zenetos et al. 2022), ultimately affecting ecosystem functioning (Tsirintanis et al. 2022; Katsanevakis et al. 2025). The properties of stressors, together with the organismal ability to respond to environmental changes– in terms of tolerance range and adaptive capacity– are key drivers of the success or failure of a species in future climate change scenarios.

Concurrent anthropogenic and environmental stressors such as overfishing, eutrophication, and temperature increase are hypothesised to trigger jellyfish outbreaks (Richardson et al. 2009; Goldstein and Steiner 2020; Sagarminaga et al. 2024). Responses of scyphozoan jellyfish populations under different environmental conditions have been investigated by measuring individual traits (e.g., asexual reproduction including polyp budding and strobilation rates, feeding rates, growth, fecundity, and survival) to assess their adaptive capacities (e.g., Treible and Condon 2019; Wang et al. 2020; Fitt et al. 2025). Nevertheless, despite increasing attention to the ecological role of cnidarian jellyfish worldwide (Bosch‐Belmar et al. 2021; Ruzicka et al. 2020; Wang et al. 2024), knowledge about their metabolism remains limited to a few species (Boero et al. 2016; Aljbour et al. 2017, 2019; Nagata et al. 2024). Studies on the effects of temperature on jellyfish metabolism have reported contrasting results, ranging from no effect (Malej 1989; Purcell et al. 2010; Kuplik et al. 2021) to significant metabolic changes (e.g., *Aurelia* sp.: Møller and Riisgård 2007; Gambill and Peck 2014; Höhn et al. 2017; Frolova and Miglietta 2020).

The Mediterranean Sea, a semi‐enclosed basin characterized by a high proportion of endemic species, has become a hotspot for non‐native species introduced by different pathways including the dispersal facilitated by the opening of the Suez Canal, fouling from maritime traffic, ballast water discharge, and aquarium trades (e.g., Galil et al. 1990; Deidun et al. 2018; Zenetos et al. 2022; Toso et al. 2024; Furfaro et al. 2025). Among them, the so‐called upside‐down jellyfish *Cassiopea andromeda* (Forskål, 1775), first described in the Red Sea, was reported for the first time in the Mediterranean Sea over 120 years ago (Maas 1903), a few decades after the opening of the Suez Canal in 1869. Now this species is enlarging its distribution range and abundance, from the Eastern to the Central and Western Mediterranean (Galil et al. 1990; Çevik et al. 2006; Schembri et al. 2010; Cillari et al. 2018; Ramos‐Pérez et al. 2025). As for other Rhizostomeae species (sub‐order Kolphophora), *C. andromeda* has a mixotrophic, intracellular symbiotic relationship with dinoflagellates (zooxanthellae, family Symbiodiniaceae) which is acquired horizontally from the environment at the polyp stage (Djeghri et al. 2019). This symbiosis influences the holobiont biochemical composition, nutrition strategy, and metabolism (Mammone et al. 2021; Nagata et al. 2024; De Domenico et al. 2025). Upside‐down jellyfish species live in shallow and sheltered coastal areas, where rapid shifts in abiotic conditions may be observed (Holland et al. 2004). In the Red Sea, *C. andromeda* medusa occur in mangroves habitats and other shallow‐water areas (Mergner and Svoboda 1977; Niggl and Wild 2010). In the Mediterranean Sea and Atlantic Ocean, it has been found in harbours (Galil et al. 1990; Cillari et al. 2018; De Rinaldis et al. 2021), saltmarshes (Deidun et al. 2018), enclosed aquaculture facilities (Morandini et al. 2017; Thé et al. 2020; Gueroun et al. 2024), and other eutrophicated confined environments or canals (Çevik et al. 2006; Ramos‐Pérez et al. 2025).

Despite the crucial role of the polyp stage in controlling the outbreak dynamics of most medusozoan jellyfish populations (Kingsford et al. 2000; Boero et al. 2002; Hubot et al. 2017), the scyphistoma ecophysiology has been relatively unexplored, and its distribution and vertical zonation in nature are poorly known (Frolova and Miglietta 2020). So far, the *Cassiopea* spp. polyps' responses (e.g., survival, asexual reproduction) to environmental changes have been addressed in mesocosm experiments (e.g., Rahat and Adar 1980; Fitt and Costley 1998; Klein et al. 2016, 2017; Goldstein and Steiner 2020). Recent studies on *C. andromeda* ecophysiology have focused on the ephyrae (Banha et al. 2020) and medusa stages (Aljbour et al. 2017, 2019, 2023; Mammone et al. 2021; Aljbour and Agustí 2024). However, the eco‐physiological responses to increasing temperatures and the species' thermal tolerance range have not been investigated yet, leaving uncertainties on the potential geographic enlargement and possible impacts of *C. andromeda* jellyfish in a Mediterranean Sea warming scenario.

In this study, the polyp stage of *C. andromeda* has been experimentally investigated to identify its thermal tolerance range using respiration rate as a metabolic proxy. This trait‐based approach has enabled the definition of the species' fundamental thermal niche, optimal temperature, and critical tolerance thresholds for growth and survival (critical pejus ranges, Sokolova et al. 2012). By integrating outputs from this mechanistic approach within a modelling framework, metabolic‐based maps were generated to foresee the potential thermal habitat suitability of the species along the Mediterranean coasts under present and future greenhouse gas emission scenarios, according to the Representative Concentration Pathways projections (RCP 4.5 and 8.5 scenarios for 2050, IPCC 2021).

## 2 Materials and Methods

### 2.1 *Cassiopea andromeda* Thermal Range and Potential Ecological Implications

To define the temperature ranges to be tested experimentally for *C. andromeda* polyps, an extensive global review on the species occurrence was performed. Data on *C. andromeda* were extracted from different sources of information: (i) peer‐reviewed literature extracted from SCOPUS, Google Scholar, and Web of Science and (ii) online occurrences database (see Table S1). Retrieved information (literature reference, sampling area, location, country, depth (m), year, latitude, longitude, temperature (°C); Table S1) dealt mostly with records of the medusa stage, because in situ observations of *C. andromeda* polyps are sporadic (Prasade et al. 2016; Morandini et al. 2017). The benthic habitat of the upside‐down jellyfish, almost exclusively recorded laying on the sea bottom, supports the hypothesis that they live in the same environment where the polyp stage occurs in cryptic conditions. In addition, to discuss the potential impact of *C. andromeda* proliferation, a literature review was conducted by using the keywords (“ *Cassiopea andromeda* ” OR “*Cassiopea*”) AND impact* across SCOPUS, Google Scholar, and Web of Science databases. Although the experimental focus of this study is on *C. andromeda* , the review includes studies in all congeneric species. This broader methodology was adopted due to the limited literature available on *C. andromeda* and the still dubious identifications of the species worldwide (Jarms and Morandini 2019). Given the ecological and functional similarities among *Cassiopea* species, as they often occupy comparable ecological niches (Morejón‐Arrojo and Rodriguez‐Viera 2023; Morejón‐Arrojo et al. 2025), genus‐level observations were considered relevant and informative for evaluating the potential consequences of *C. andromeda* expansion in non‐native habitats.

### 2.2 Aquarium Maintenance and Acclimation of Polyps

#### 2.2.1 Maintenance

The experiments were carried out with a second‐generation polyp culture of *C. andromeda* , originally from the Mediterranean Sea and maintained at the Genova Aquarium (Italy). The species was identified as *C. andromeda* , using 16S DNA barcoding. The specimens used for the experiment were kept in glass aquaria with filtered sea water (Whatman GF/F filters, 0.7 μm), at a constant temperature of 24°C and 35 salinity, and daily fed with the first stage nauplii of *Artemia salina* .

#### 2.2.2 Temperature Treatment and Acclimation

To investigate the thermal tolerance of the species, a total of 210 polyps were used (mean dry weight of two polyps 1.210 ± 0.007 mg). Using a plastic micropipette, the polyps were gently detached from the aquarium walls, transferred into a glass bowl, and observed at the stereomicroscope until re‐extension of the polyp column and tentacles to confirm healthy conditions. For each temperature treatment, 14 polyps were placed together in a single 1 L tank filled with freshly filtered seawater, maintained at 24°C and a salinity of 35. Although polyps within each treatment group were exposed to the same thermal conditions, they were spatially separated within the tank to minimize physical interactions. Individuals were starved for 24 h prior to the onset of temperature acclimation. Based on available knowledge of the species' potential thermal range (see references in Table S1), polyp metabolic experiments were launched at 15 different temperatures, namely at 12, 14, 16, 18, 20, 22, 24, 26, 28, 30, 32, 34, 36, 38, and 40 (°C). In each experimental group, temperature was increased or decreased to the next level with the use of a thermal controlled bath Grant Optima 150 (Prusina et al. 2014; Bosch‐Belmar et al. 2022; Marchessaux et al. 2022). Starting from the acclimatized water temperature, the ramp to reach the target temperature was modified at a rate of 1°C every 2 h (~0.008°C min^−1^) to avoid polyp strobilation and/or budding due to stress. Building on established approaches (Montalto et al. 2017; Bosch‐Belmar et al. 2021; Shirokova et al. 2025), a more gradual temperature increase was applied to determine the thermal tolerance limits of polyps under standardized laboratory conditions while minimizing potential confounding stress responses. After the target experimental temperatures were reached, all individuals were held at those temperatures for an additional 24 h to ensure consistent short‐term acclimation before the start of the respiration measurement.

### 2.3 Metabolic Traits Measurements and Thermal Performance Curve

#### 2.3.1 Respiration Rate and Gross Primary Production

The measurement of oxygen consumption as function of temperature was performed with two oxygen meters, PyroScience Fire‐sting O_2_, each equipped with four optodes. Oxygen consumption was measured on seven respiration chambers of 0.8 mL each, filled with filtered seawater (GF/F filters, 0.7 μm). After being transferred from their 1 L aquaria to the respiration chambers, polyps underwent a 45 min acclimation period. This step aimed to minimize handling stress before the oxygen consumption measurements, with two polyps in each chamber (*N* = 7 chambers) and one control (without polyps) for each target experimental temperature. Using two polyps per chamber ensured a sufficiently strong oxygen signal for accurate measurement, as preliminary tests showed that oxygen changes from a single polyp were too subtle to reliably detected. For each experimental temperature, continuous oxygen measurements were conducted over a two‐hour period, of which 1 h was under dark and 1 h was under light conditions, to assess oxygen consumption and production, respectively. Both temperature and oxygen concentration were recorded continuously every second throughout the entire incubation period. To prevent oxygen stratification in the water, every 15 min, the respiration chambers were gently agitated. The experimental and control chambers were filled with filtered seawater and processed under identical conditions to the acclimation ramp. Control chambers, which did not contain polyps, consistently showed no measurable background oxygen consumption, confirming that all observed changes in oxygen concentration in the experimental chambers were attributable to the metabolic activity of the polyps and their associated holobiont. After measurements, the wet weight (WW, mg) of each replicate (i.e., two polyps) was obtained with an analytical balance, as well as the dry weight (DW, mg) after drying each replicate at 60°C for 24 h in an oven. The respiration rate (RR) was calculated as:

$$\mathrm{RR}=\frac{\beta_{\left[O2\right]}*t*\mathrm{Vol}}{\mathrm{DW}}\left(\mathrm{mg}O_{2}\;h^{-1}g^{-1}\right)$$

where β_[O2]_*t*Vol represents the slope of the measured oxygen concentration in the respiration chambers (β_[O2]_) standardized per time unit (*t, s*) and per the volume of the respiration chambers (Vol, L). To account for the effect of body mass on RR, values were expressed relative to the individuals' DW.

The RR accounts for the oxygen concentration retrieved under dark conditions. The Net Primary Production (NPP) was calculated following the same equation and accounted for the oxygen produced by the symbiont considering the host and zooxanthellae respiration process (measurements performed under light condition). The Gross Primary Production (GPP) represents the total net amount of oxygen produced through photosynthesis by the zooxanthellae symbiont (*Symbiodinium* sp.). Assuming that the rate of RR is constant through the daily cycle, the rate of GPP was estimated as: GPP = NPP + |RR|.

#### 2.3.2 Thermal Performance Curve (TPC)

Based on the individual RR and GPP obtained experimentally at each treatment level, the thermal performance curve of the species was constructed. According to (Padfield et al. 2021) pipeline, a total of 24 non‐linear least‐squares models were fitted and compared with the use of the “rTPC” package (Padfield and O'Sullivan 2021) in RStudio software (v. 4.3.2). The best model was selected based on the lowest Akaike's information criterion (AIC) score. To calculate the uncertainty in the best model and optimal confidence intervals, bootstrapping was used to determine the 95% prediction limits. The *optimum* temperature range (i.e., the maximum aerobic scope) and the critical *pejus* range (where the aerobic scope starts to be impaired due to elevated metabolic cost, i.e., critical maximum and minimum temperatures) (Sokolova et al. 2012) have been identified according to Pawar 2018 (Kontopoulos et al. 2018), recognized here as the best model.

Pawar 2018 is a non‐linear regression model describing the TPC of an organism based on enzymatic response to temperatures, as:

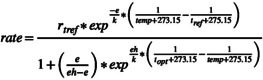

where: *rate* is RR and GPP, *temp* is temperature (°C), *r*_*tref*_ represents the real rate performance at a reference tested temperature, *e* is the enzyme activation energy (eV), *eh* is the high temperature de‐activation energy (eV), *t*_*opt*_ is the *optimum* temperature, *t*_*ref*_ = 15°C is the temperature at which the respiration is not inactivated by high temperatures and is provided by the model, and *k* = 8.62*10^−5^ (eV K^−1^) is the Boltzmann constant (Kontopoulos et al. 2018). The terms of the Pawar non‐linear regression according to the RR were: *r*_*tref*_ = 0.12°C, *e* = 0.6 eV, *eh* = 14.4 eV, *t*_*opt*_ = 35.7°C, and the terms for GPP were: *r*_*tref*_ = 0.28°C, *e* = 0.6 eV, *eh* = 12.3 eV, *t*_*opt*_ = 35.4°C.

### 2.4 Current and Future Thermal Habitat Suitability Maps

The final parameters of the TPC model were used to develop current and future thermal habitat suitability (THS) maps based on species metabolism functioning. In doing so, the experimentally obtained values of RR were transformed into probability by dividing the respiration rate value at the tested temperature (RR_Temp_) by the RR at the *optimum* temperature predicted by the model (RR_Opt_) (Bosch‐Belmar et al. 2022). These normalized values represent THS scores, ranging from 0 to 1. Values close to 0 reflect sub‐optimal thermal conditions and values approaching 1 indicate optimal environmental conditions for metabolic activity. Thus, THS values represent the relative physiological favorability of environmental temperatures for polyp persistence.

The THS values were then applied to produce the THS maps with Mediterranean Sea mean monthly temperature raster files representing the current (2021–2023) and future (2050) climatic conditions. As *C. andromeda* is a coastal species, the analyses were limited to a bathymetry range of 0–50 m. The environmental data for the current Sea Surface Temperature (SST) were downloaded from the Copernicus Marine Service Information (www.copernicus.eu), and raster layers were upscaled to a resolution of 1 km^2^. In the Mediterranean Sea, highest temperatures occur between July and September (mean SST 27°C ± 2°C), and the lowest are observed from December to March (mean SST 13°C ± 2°C) (Pisano et al. 2020). Maps for future scenarios were created with modelling projections forced by Representative Concentration Pathways RCP 4.5 and 8.5 (IPCC 2021). The RCP 4.5 scenario represents a future with moderate greenhouse gas mitigation efforts, leading to a stabilization of emissions by mid‐century, whereas the RCP 8.5 scenario assumes a high‐emission trajectory with continued fossil fuel reliance and minimal mitigation, resulting in more severe climatic changes.

## 3 Results

### 3.1 *Cassiopea andromeda* Thermal Ranges

**FIGURE 1:**
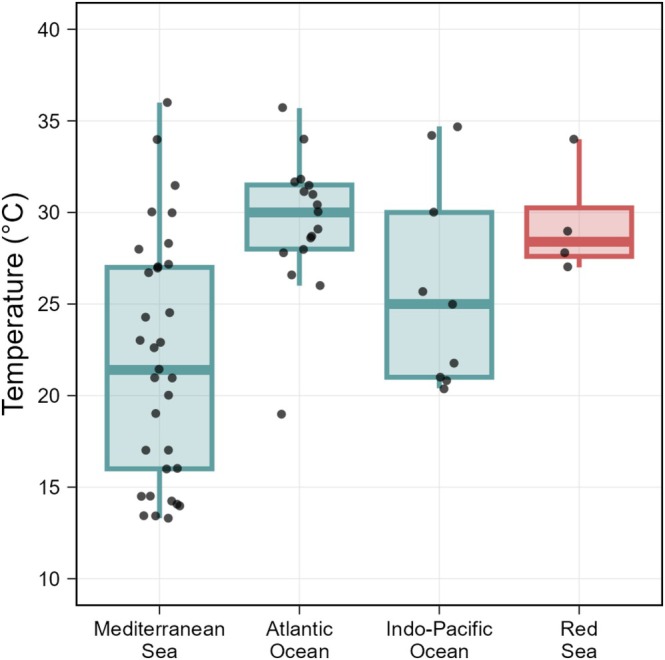
Ranges of temperature observed for *Cassiopea andromeda* in the non‐ native areas (blue) of the Mediterranean Sea (*N* = 33), Atlantic Ocean (*N* = 17), and Indo‐Pacific Ocean (*N* = 9) and in its native area (Red Sea in red; *N* = 4) obtained from the literature and online data bases (Table S1).

*Cassiopea andromeda* has been observed across a broad temperature range within its current geographic distribution at depths from 0 to 25 m, with a single observation reported at 96 m (see references in Table S1). In Red Sea mangrove habitats, *C. andromeda* thrives within a narrow temperature range from 27.0°C to 34.0°C (Figure 1). In the Mediterranean Sea, the thermal range is wider, ranging from 13.0°C to 36.0°C. In the Atlantic and Indo‐Pacific Oceans, the species has been observed in temperature ranges from 19.0°C to 35.7°C and 20.8°C to 34.7°C, respectively (Figure 1, Table S1). This information supported the selection of a range of temperatures between 12°C and 40°C for the experimental studies.

### 3.2 *Cassiopea andromeda* Potential Ecological Implications

The compiled literature reveals a complex ecological profile for *Cassiopea* spp., with potential impacts ranging from disadvantageous to beneficial, depending on the environmental context and the species' ecological interactions (Table 1).

**TABLE 1:** Summary of potential ecological and economic impacts associated with *Cassiopea* spp. occurrence reported in the literature.

General type of impact	Details	Impact (Positive/Negative)	References
Oxygen depletion	Night depletion of dissolved oxygen at the sediment–water interface.	Negative	Verde and McCloskey (1998)
Competition with benthic fauna	Dense jellyfish aggregations may let demersal fish avoid these areas and cassiosomes could be lethal. Competition for particulate organic matter with filter feeding consumers.	Negative	Stoner et al. (2011, 2022); Stoner, Yeager, and Layman (2014)
Competition for light with seagrass and mechanic disturbance	Compete for light with benthic flora, modifying seagrass structure and biomass. Disturb seagrass shoots by a mechanic action.	Negative	Stoner et al. (2011) Stoner, Yeager, and Layman (2014)
Alteration of species composition	Compete with native species and act as a vector for non‐native species.	Negative	Weeks et al. (2019)
Alteration of biogeochemical cycles	Increases ammonium uptake, reduces nitrate uptake, promotes nitrogen recycling, limits denitrification.	Negative and Positive	Welsh et al. (2009); Jantzen et al. (2010); Niggl and Wild (2010); Stoner et al. (2011); Zarnoch et al. (2020); Durieux et al. (2021)
Ecosystem engineer species	The bell mechanic action in shallow coastal habitats ensure the vertical mixing of water and facilitate benthic‐pelagic coupling and primary production in oligotrophic coral reefs.	Positive	Niggl and Wild (2010); Jantzen et al. (2010); Durieux et al. (2021)
Bioindicator of pollutants	Accumulates microplastic, pesticides, herbicides, heavy metals, dissolved inorganic phosphates.	Positive	Templeman and Kingsford (2015); Epstein et al. (2016); Klein et al. (2016); McKenzie et al. (2020); Templeman et al. (2021); Béziat and Kunzmann (2022); Caldwell et al. (2023)
Habitat former and food source	Provides shelter and feeding ground for many marine organisms and possibly protects them from grazing activities.	Positive	Niggl and Wild 2010
Blue Growth potential	Source of sustainable bioactive compounds for suitable use in pharmaceutical, food, and nutraceutical sectors.	Positive	De Rinaldis et al. 2021; De Domenico et al. (2023); De Domenico et al. 2025)

### 3.3 Respiration Rate and Gross Primary Production

**FIGURE 2:**
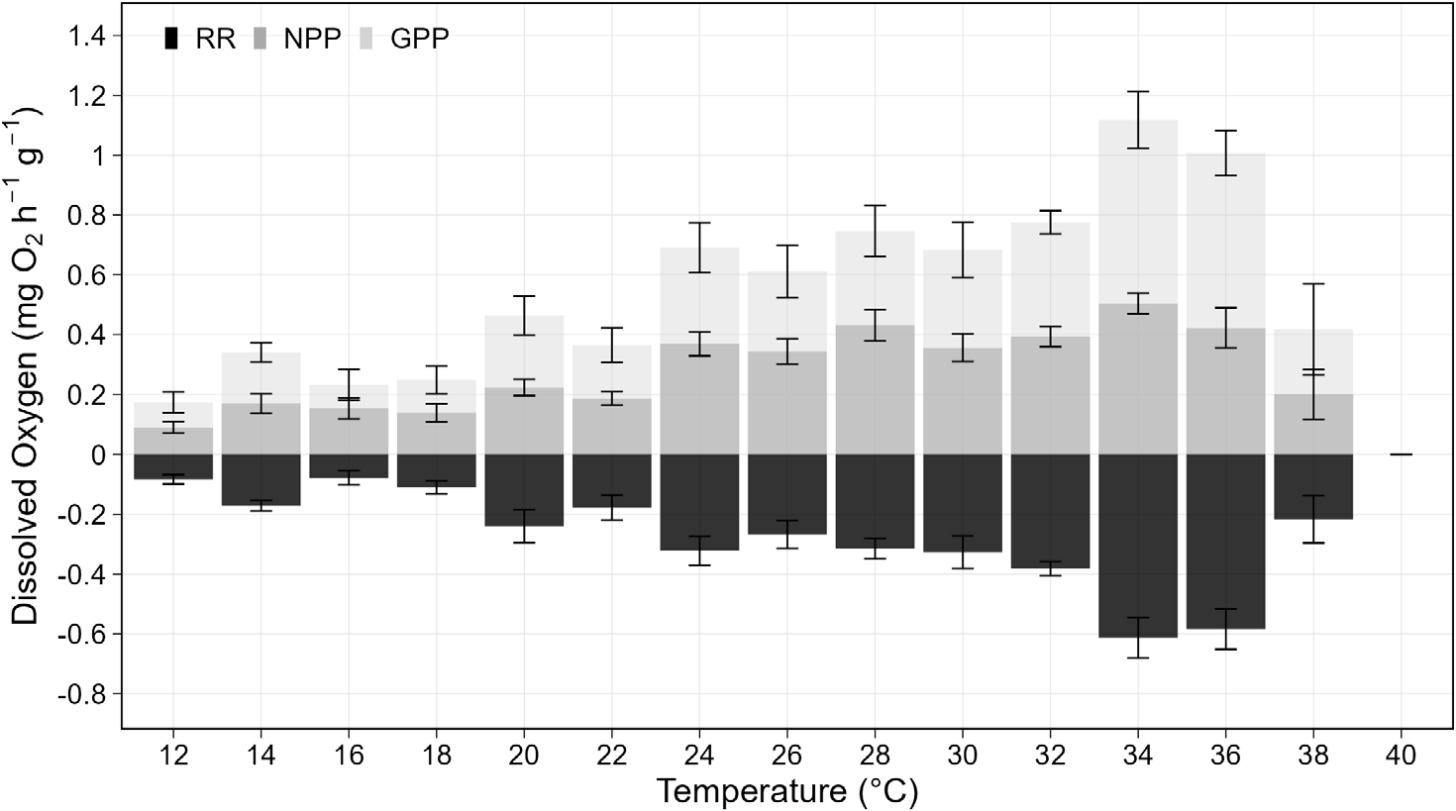
Dissolved Oxygen (mg O_2_ h^−1^ g^−1^) measured under laboratory conditions for the Respiration Rate (RR, black), Net Primary Production (NPP, medium grey), and Gross Primary Production (GPP, light grey) of *Cassiopea andromeda* polyps as function of temperature (°C). Error bars indicate the standard error.

Polyps maintained effective metabolic activity in terms of both RR and NPP across the 12°C to 38°C range (Figure 2, Table S2). Between 12°C and 38°C, the RR varied from 0.08 ± 0.02 mg O_2_ h^−1^ g^−1^ at 12°C to 0.22 ± 0.08 mg O_2_ h^−1^ g^−1^ at 38°C, with a maximum of 0.61 ± 0.07 mg O_2_ h^−1^ g^−1^ at 34°C, while NPP rose from 0.09 ± 0.02 mg O_2_ h^−1^ g^−1^ to 0.20 ± 0.08 mg O_2_ h^−1^ g^−1^ over the same interval, with a maximum of 0.50 ± 0.03 mg O_2_ h^−1^ g^−1^ at 34°C, reflecting a general trend of enhanced metabolic activity with temperature. RR and NPP peaked at 34°C, and at 36°C the values decreased to 0.58 ± 0.07 mg O_2_ h^−1^ g^−1^ for RR and 0.42 ± 0.07 mg O_2_ h^−1^ g^−1^ for NPP. Beyond 36°C, RR dropped drastically (0.22 ± 0.08 mg O_2_ h^−1^ g^−1^ at 38°C), likely due to physiological damage or thermal stress, as 20% mortality was observed.

NPP remained higher than RR up to approximately 32°C (Table S2). Above this threshold, NPP fell below RR, indicating that respiration surpassed photosynthetic production (Figure 2). Outside the thermal range of 20°C–38°C, NPP exhibited a significant reduction, with oxygen concentrations below 0.19 mg O_2_ h^−1^ g^−1^ (Figure 2, Table S2). GPP followed a similar pattern to RR and NPP, gradually rising with temperature and peaking at 34°C (1.12 ± 0.09 mg O_2_ h^−1^ g^−1^). At 38°C, GPP also had a significant decline (0.42 ± 0.15 mg O_2_ h^−1^ g^−1^), signaling a potential lower photosynthetic activity of the symbiont. Except for the temperatures of 12°C and 14°C, in which no mortality was observed, at 16°C, 18°C, and 38°C, 20% of polyps did not survive, while at 40°C, mortality reached 100% (Figure 2).

### 3.4 Thermal Performance Curve

**FIGURE 3:**
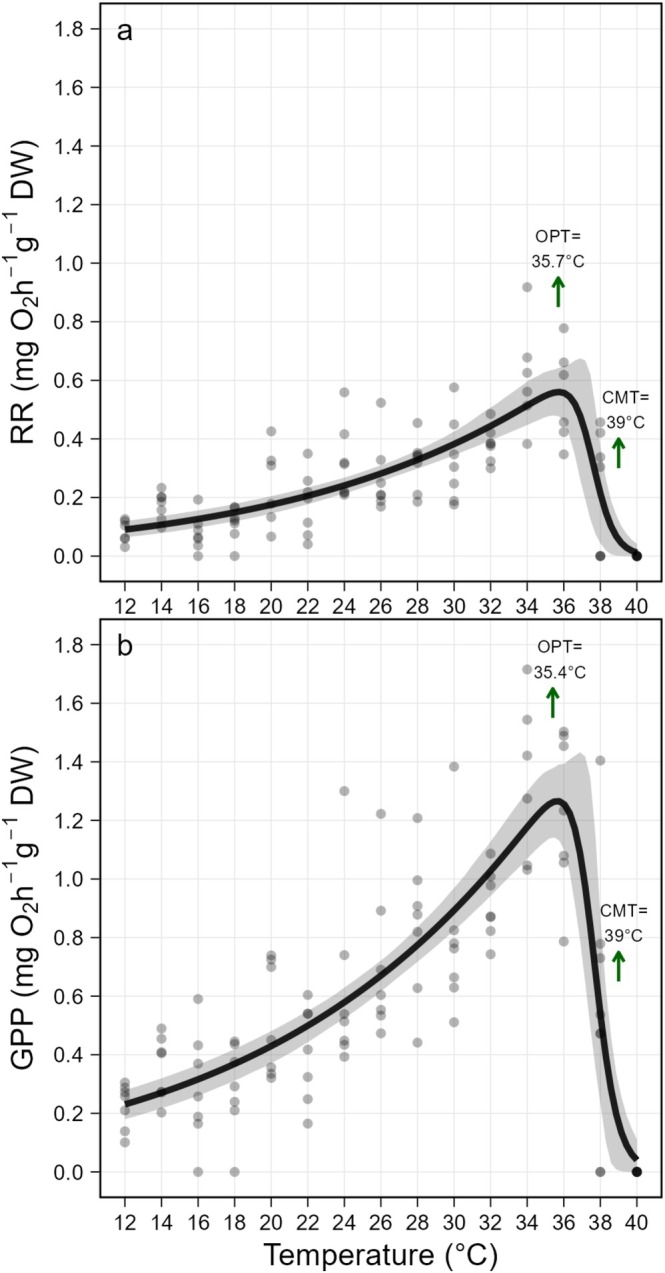
Thermal performance curve of *Cassiopea andromeda* polyps according to the Pawar model (a) based on the Respiration Rate (RR, mg O_2_ h^−1^ g^−1^) and (b) based on the Gross Primary Production (GPP, mg O_2_ h^−1^ g^−1^) as function of temperature (°C). Dots represent each individual respiration rate measured, and darker dots represent overlaps of these measurements. The grey band represents the 95% confidence interval. Optimum Temperature (OPT) and Critical Maximum Temperature (CMT) are indicated with green arrows.

The TPCs of *C. andromeda* polyps, in terms of RR (Figure 3a) and GPP (Figure 3b), were fitted with the Pawar 2018 model (Kontopoulos et al. 2018). The species TPCs exhibited a left‐skewed gaussian shape across the tested temperature range, with a gradual decline in RR and GPP at low and high temperatures (Figure 3a,b). The RR and GPP thermal optima were observed at 35.7°C (Figure 3a) and 35.4°C (Figure 3b), respectively. In both RR and GPP, the predicted critical maximum temperature was 39.0°C and the minimum was extrapolated as 6.4°C.

### 3.5 Temperature Habitat Suitability Maps

**FIGURE 4:**
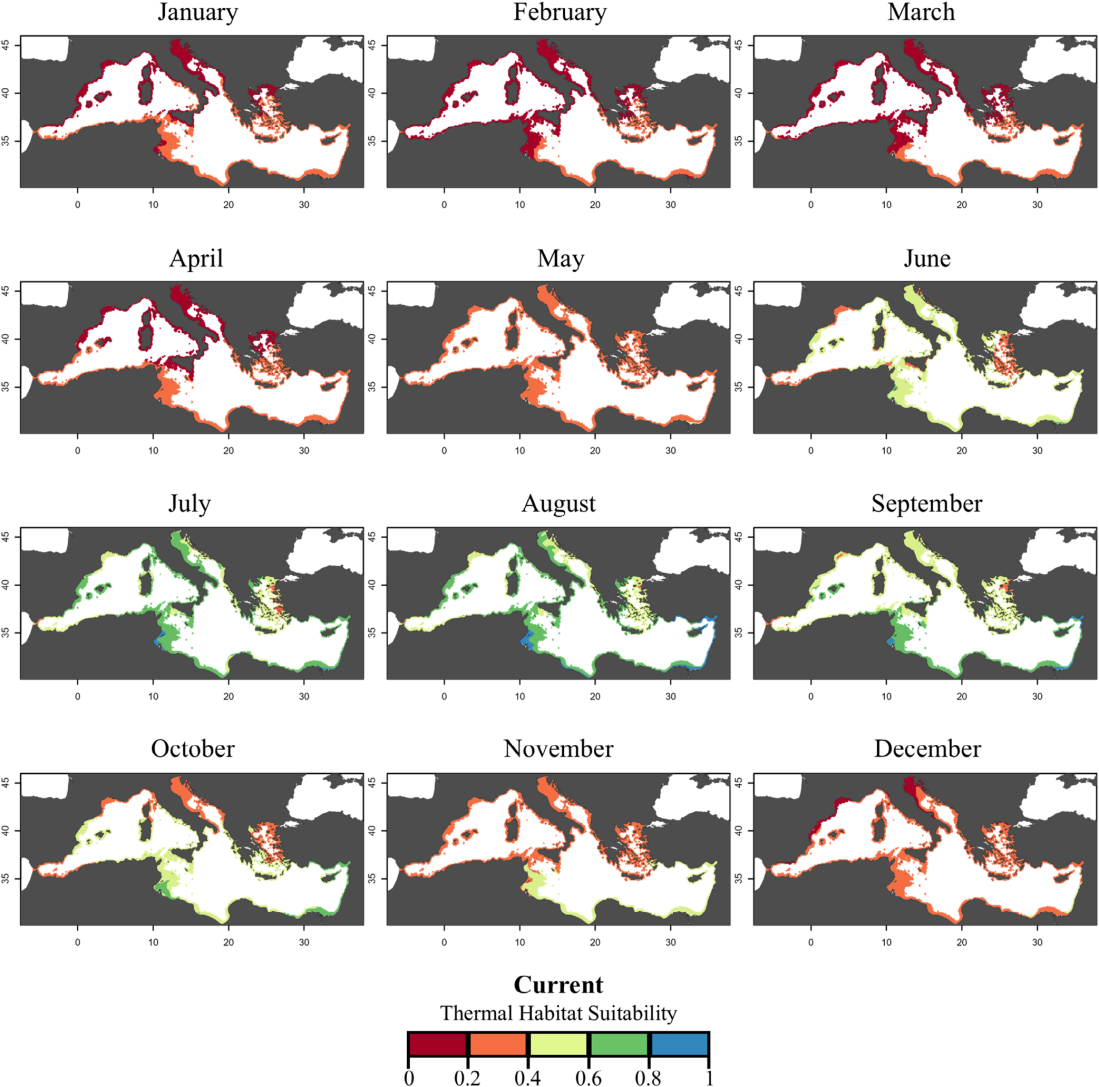
Monthly thermal habitat suitability maps (as probability) for *Cassiopea andromeda* polyps in the Mediterranean Sea based on the Respiration Rate for current climatic conditions (2021–2023). Different colors represent the THS scale and correspond to: *Optimum* temperature range (green and blue), lower and upper *pejus* ranges (yellow and orange), and *pessimum* range (red).

The current thermal habitat suitability (THS) for *C. andromeda* in the Mediterranean Sea showed a monthly variability across different regions of the basin (Figure 4, Table S3). From January to March, THS remained low overall, with most areas falling within the 0.0–0.2 (59.43% ± 13.74%) and 0.2–0.4 (46.93% ± 13.74%) classes. Between April and June, a gradual increase in THS was observed, particularly along the southern and central Mediterranean coasts, as well as in the Aegean Sea, northern Adriatic Sea, and central Tyrrhenian Sea. In May, the class 0.2–0.4 represented 97.82%, whilst in June it was reduced to 18.73% and the class 0.4–0.6 rose to 79.10%. Suitability reached the maximum during the summer months, with July and August displaying the highest proportions: 29.43% ± 0.73% in the 0.4–0.6 class, and 62.90% ± 2.67% in the 0.6–0.8 class, respectively. Notably, August recorded the highest percentage (8.89%) throughout the year in the 0.8–1.0 class. From September onwards, a progressive decline in THS was observed. In September, suitability was still relatively high, with the 0.4–0.6 class dominating (57.49%), followed by 36.09% in the 0.6–0.8 class and 3.17% in the 0.8–1.0 class. By October, moderate suitability persisted, with 54.06% in the 0.4–0.6 class and 11.64% in the 0.6–0.8 class, while lower classes began to expand (33.51% in the 0.2–0.4 class). In November, the decline accelerated, with 69.93% of the basin within the 0.2–0.4 class and 28.88% in the 0.4–0.6 class. By December, low suitability dominated, with 85.52% of the basin in the 0.2–0.4 class and 11.71% in the 0.0–0.2 class.

**FIGURE 5:**
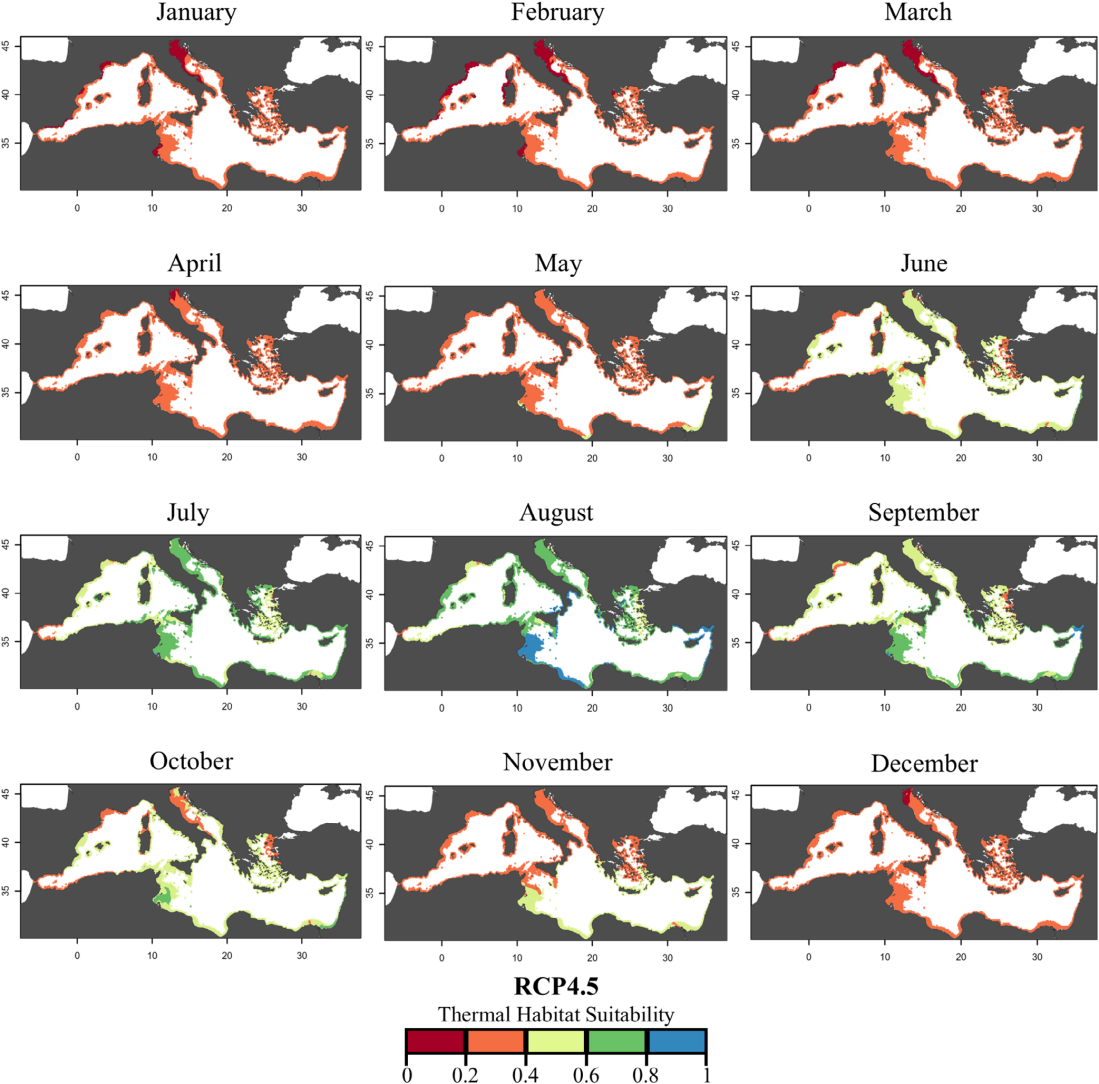
Monthly thermal habitat suitability maps (as probability) under future climate change conditions (RCP 4.5, 2050) predicted for *Cassiopea andromeda* polyps in the Mediterranean Sea based on the Respiration Rate. Different colors represent the THS scale and correspond to: *Optimum* temperature range (green and blue), lower and upper *pejus* ranges (yellow and orange), and *pessimum* range (red).

Compared with the current scenario, a spatial increase in THS for *C. andromeda* was predicted under the RCP 4.5 scenario by 2050 (Figure 5, Table S3). From January to April, although suitability remained generally low, a clear shift from the 0.0–0.2 class to the 0.2–0.4 class was observed, with monthly increases of 31.41%, 48.55%, 54.86%, and 43.24%, respectively. In May, the class 0.4–0.6 further increased by 3.49% on the eastern Mediterranean coastline. During summer (June–August), more pronounced redistributions occurred. In June, the 0.2–0.4 class expanded by 3.41%, and in July, the 0.4–0.6 class increased by 6.77%. Interestingly, in August, the 0.8–1.0 class exhibited an increase of 14.28%, corresponding to 23.17% of the coastal area of the southern central Mediterranean Sea. In September, the 0.4–0.6 class prevailed (60.19%), and the 0.6–0.8 class was the second most expanded one (31.73%). In October, the expansion of the 0.4–0.6 class was 16.71% and persisted until November (5.30%) in the central Mediterranean. At the end of the year, the suitability mostly decreased, with the 0.2–0.4 class dominating the coastline in December (95.83%).

**FIGURE 6:**
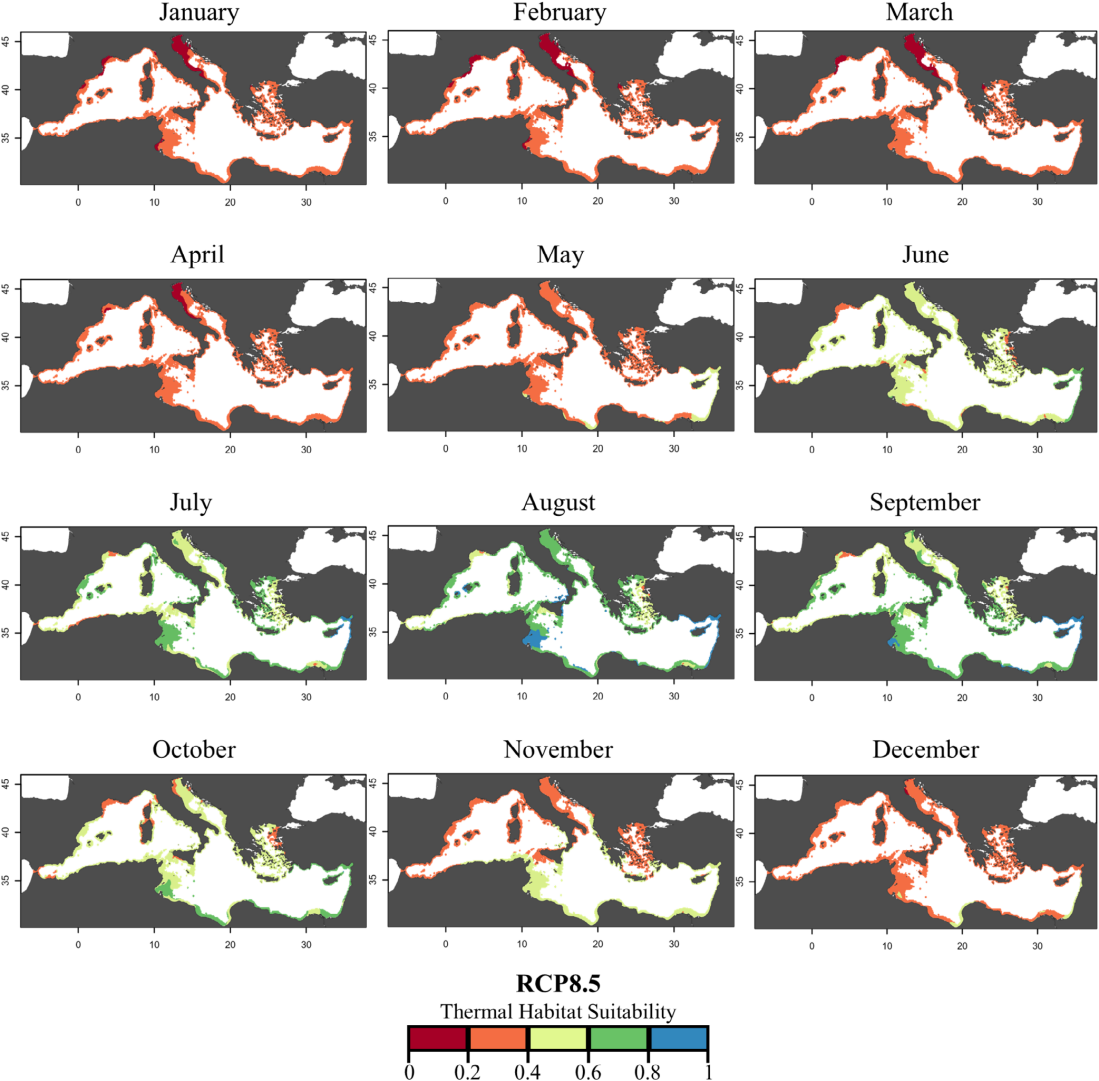
Monthly thermal habitat suitability maps (as probability) under future climatic conditions (RCP 8.5, 2050) predicted for *Cassiopea andromeda* polyps in the Mediterranean Sea based on the Respiration Rate. Different colors represent the THS scale and correspond to: *Optimum* temperature range (green and blue), lower and upper *pejus* ranges (yellow and orange), and *pessimum* range (red).

Unlike the RCP 4.5 scenario, projections under RCP 8.5 indicated more pronounced changes in THS for *C. andromeda* by 2050 compared with current conditions (Figure 6, Table S3). From January to April, a shift towards the 0.2–0.4 class was observed, with monthly increases of 32.42%, 52.11%, 55.20%, and 39.06%, respectively. In May, an increase in suitability was detected, with a 7.05% expansion in the 0.4–0.6 class. In June, a slight increase (6.16%) in the 0.4–0.6 class and a 3.02% increase of the 0.6–0.8 class were recorded in the northern Adriatic and central Mediterranean Sea. July showed a marked increase (19.33%) in the 0.4–0.6 class (more than double that under RCP 4.5), while in August, suitability increased by 6.67% in the 0.6–0.8 class and 5.43% in the 0.8–1.0 class (corresponding to the 67.68% and 14.32% of coastline, respectively). Both in present and future scenarios, August was the month with the hugest spatial extension of the optimal suitable conditions for the species. Interestingly, September had a 20.75% increase of the 0.6–0.8 class and a 3.26% increase of the 0.8–1 class, with the latter duplicating the covered coastline area compared with current conditions (3.17% of the area in the current scenario, and 6.43% in the RCP 8.5 projection). Albeit in October, the 0.6–0.8 class still increased (7.01%), the highest increment occurred in the 0.4–0.6 class (by 15.32%), which was sustained until November (15.67% of increase). In December, suitability gradually declined compared with previous months, with a strong dominance of the 0.2–0.4 class (93.33% of the coastline).

## 4 Discussion

The geographical distribution of the medusa stage suggests that *C. andromeda* exhibits a high degree of adaptive plasticity that is consistent with its presence in a variety of marine habitats such as the Red Sea (Holland et al. 2004), the Mediterranean Sea (Galil et al. 1990; Schembri et al. 2010; Cillari et al. 2018; Ramos‐Pérez et al. 2025), the Atlantic Ocean (Thé et al. 2020; Muffett and Miglietta 2023; Gueroun et al. 2024), and the Indo‐Pacific Ocean (Holland et al. 2004; Karunarathne et al. 2020), showing a wide realized thermal niche between 13°C and 36°C. This adaptability was further experimentally supported here, showing that the polyps' thermal tolerance presents a wide window and resistance from 12°C to 39°C.

The left‐skewed Gaussian shape identified for the polyps with the TPCs is typical of high‐temperature specialists (Angilletta et al. 2010), which often corresponds to invasive species patterns (Simon and Amarasekare 2024). The thermal optimum at 35.4°C (GPP)–35.7°C (RR) found in this study might be an adaptation to its tropical habitat as mangroves and seagrass beds, which are subject to intense temperature fluctuations (Ohdera et al. 2018). Notably, RR escalated more rapidly than NPP, suggesting a progressive energy imbalance at elevated temperatures. The polyp's tolerance is expected to be wider than the ephyra and medusa due to their ability to survive under adverse environmental conditions through a resting stage with a low metabolic demand (Boero et al. 2008). Field and laboratory studies in the congeneric species *C. xamachana* showed that polyps were more sensitive to low temperatures than medusae, with polyps often not observed over winter, whereas medusae survived (Fitt and Costley 1998). Meanwhile, in situ observations on *C. andromeda* populations indicate that medusae appear seasonally in the Mediterranean Sea (e.g., Schembri et al. 2010 and Deidun et al. 2018 in Malta; Özgür and Öztürk 2008 in Turkey; Mammone et al. (2023); Fumarola *pers. obs*. in “La Cala”, Sicily, Italy) and Atlantic Ocean (Thé et al. 2020). This intermittent presence suggests that long‐term population persistence is primarily sustained by the polyp stage. Our results also suggest that polyps of *C. andromeda* are capable of surviving at lower temperatures by entering a low‐metabolic‐cost state rather than dying. At low temperatures (16°C and 18°C), a mortality rate of 20% was observed. However, the survival of some polyps even at lower temperatures (12°C and 14°C) suggests mortality due to intraspecific variability in thermal tolerance (Nati et al. 2021). *Cassiopea* sp. medusae showed that they can fully acclimatise without significant oxidative stress effects at 32°C (Aljbour et al. 2018, 2019). In addition, after three consecutive thermal stress events at 33°C, neither the chlorophyll *a* concentration nor the growth of *C. andromeda* ephyrae were affected (Banha et al. 2020). These results support the identified thermal optimum in the present study. Beyond 36°C, the polyp functional machinery entered a state of thermal stress, presenting a mortality rate of 20%. Polyps of the congeneric species *C. xamachana* exhibited high mortality when exposed experimentally to heat stress of 36°C (Fitt et al. 2025). Here, a reduction of NPP at 38°C was observed, suggesting a malfunctioning of the photosynthetic systems of the symbiont or a reduction of them by the expulsion of the microalgae (Klein et al. 2017). This could be a strategy to survive in adverse conditions and rely on the heterotrophic behavior rather than the autotrophic one to avoid the production of Reactive Oxygen Species (ROS) from the symbiont (Roth 2014).

The relationship between oxygen consumption and temperature has been rarely investigated in scyphozoans, and the observed responses showed intra and interspecific variability. In *Pelagia noctiluca* and *Rhopilema nomadica*, the respiration rate was measured as a function of body mass at ambient temperatures, but no significant changes were detected (Malej 1989; Kuplik et al. 2021). Meanwhile, three different populations of *Aurelia aurita* polyps in the North Sea showed respiration responses to ambient temperature (2°C–22°C) that differed according to the geographic area, indicating an acclimation to local sea temperatures (Höhn et al. 2017). However, no information about the critical temperature limits is available for *C. andromeda* polyps. In the present study, oxygen consumption of *C. andromeda* polyps was assessed across a broad temperature range (12°C–40°C), and it included measurements at the species' critical temperature limits, providing essential information on the thresholds that define survival. These limits are key in determining the species' vulnerability to temperature fluctuations and the impacts of climate change, ultimately contributing to the development of reliable predictive models. Yet, thermal tolerance limits have been only sporadically explored in scyphozoans. For example, three different species of *Aurelia* spp. inhabiting two bathymetries of the Gulf of Mexico and one of the South China Sea revealed that the coastal species of the Gulf of Mexico had a critical maximum temperature of 37°C, suggesting its acclimation and affinity to higher temperatures (Frolova and Miglietta 2020). In this study, a critical minimum temperature of 6.4°C and a critical maximum temperature of 39.0°C were observed for *C. andromeda* polyps, supporting the thermophilic affinity of the species. Larval settlement and metamorphosis depend on specific abiotic conditions, along with the presence of certain bacteria and molecular cues (Hadfield and Paul 2001; Morejón‐Arrojo et al. 2025). Fitt and Costley (1998) showed that larval settlement in *C. xamachana* was significantly reduced at temperatures below 20°C. This pattern is consistent with our observations that reported optimal conditions at 34°C for the polyp and suggests that climate warming conditions may facilitate the geographic expansion and the establishment of *C. andromeda* in new areas in the future.

In the Mediterranean Sea, *C. andromeda* has been found in the widest range of temperatures compared to the Red Sea, the Atlantic, and the Indo‐Pacific Ocean. The expected fundamental thermal niche (12°C–36°C) of the species appears to be aligned with its realized thermal niche (Figure 1), suggesting that the species has adapted to these conditions. This indicates that the species is not only capable of tolerating a broader range of temperatures but also effectively covers its potential thermal habitat within this range. In contrast, in other marine areas like the Atlantic and Indo‐Pacific Oceans, where the species is found in warmer waters (19°C–35.7°C and 20.8°C–34.7°C, respectively), it appears to thrive within more specific temperatures, reflecting its adaptability to the local climate conditions. However, the biogeographical range where a species can survive throughout its entire ontogeny may also be influenced by local thermal acclimation (Gambill and Peck 2014; Höhn et al. 2017; Frolova and Miglietta 2020). This hypothesis, while supported by some studies, remains to be fully demonstrated. The challenge in testing this theory lies in the difficulty of locating *C. andromeda* polyps in the wild, as for other scyphozoan species (Sagarminaga et al. 2024; Schiariti et al. 2024; Schaub et al. 2025), making it difficult to assess their effective thermal limits and identify the environmental factors that trigger strobilation in the natural environment. In line with this, a limitation of the present study is that the polyps originated from a second‐generation aquarium stock maintained at a constant temperature of 24°C. This condition may not accurately represent the natural thermal variability encountered by polyps in the field. To our knowledge, polyps were only found in nature twice, attached to rocks at intertidal areas of dead coral reefs in India at 20.4°C–21.8°C (Prasade et al. 2016) and attached to artificial and natural substrates in Brazil at 26.6°C–28.7°C (Morandini et al. 2017). Nonetheless, the use of a long‐term laboratory‐reared stock allowed control for unknown environmental history and to obtain reproducible results. Despite these challenges, the adaptability of the species in different thermal conditions remains crucial for predicting how it may respond to future climate changes and potential shifts in its distribution (Molinero et al. 2008; Lynam et al. 2011; Isinibilir et al. 2024).

Based on experimentally derived thermal performance curves, the thermal habitat suitability maps suggested a potential seasonal expansion of the polyp stage fundamental thermal niche across different regions of the Mediterranean under warming scenarios (Figures 4, 5, 6, Table S3). These projections were generated using a mechanistic modelling approach that explicitly linked physiological thresholds of the polyp stage to spatial patterns of thermal suitability. Under present‐day conditions, high thermal suitability (considered as THS values ≥ 0.6) for *C. andromeda* was mostly restricted to the summer months, particularly July and August. Meanwhile, future climatic scenarios projected a broader spatial and temporal window of optimal conditions. Under RCP 4.5, and in less extension in the RCP 8.5, areas with high suitability greatly increased in August. Under RCP 8.5, the temporary window of high suitability widened from June until October. Notably, both RCP 4.5 and RCP 8.5 scenarios projected a marked increase in the 0.2–0.4 class from January to April, creating a more favorable period even during winter. This expansion suggests a considerable increase in the duration of suitable conditions for the species' survival, growth, and potential successful spread. The projected thermal expansion appeared more pronounced in the northern and western Mediterranean Sea, indicating the possibility of a future geographical range shift. This expansion is likely to occur both horizontally, across similar depth zones, and vertically, as warming affects deeper water layers including the thermocline (Boero et al. 2016; Lee et al. 2024). Furthermore, the species' tolerance to varying light conditions may facilitate its establishment in new habitats (Mammone et al. 2021).

In addition to spatial changes, rising sea temperatures may influence the timing of key life history events. In particular, an extended strobilation period is likely, as this process in *C. andromeda* is typically triggered when temperatures exceed 24°C (Hofmann et al. 1978; Rahat and Adar 1980; Hofmann and Kremer 1981), and may continue until physiological limits are reached (Belmaker et al. 2013). Similarly, warm temperature strobilation patterns have been observed in other rhizostome species such as *Cephea cephea* , *Cotylorhiza tuberculata* , *Lobonema lucerna*, *Nemopilema nomurai*, *Phyllorhiza punctata* , *Rhopilema esculentum* , *Rhopilema octopus*, *Rhopilema verrilli* , and *Stomolophus* sp. 2 (Schiariti et al. 2024). Moreover, temperature‐dependent patterns have been observed in native Mediterranean jellyfish species. For example, *Rhizostoma pulmo* blooms exhibited an enhanced magnitude, frequency, and duration in recent decades, concurrently with positive temperature anomalies (Leoni et al. 2021). The early life stages of *C. tuberculata* appear to be controlled by thermal conditions and determine the interannual presence in the marine environment (Prieto et al. 2010) as well as the cubomedusa *Carybdea marsupialis* (Canepa et al. 2017).

While to date no direct negative impacts have been demonstrated for *C. andromeda* in the Mediterranean, the species' proliferation raises important questions about its potential ecological and socio‐economic consequences (Sagarminaga et al. 2024; Table 1). *Cassiopea* spp. could alter sediment dynamics, reduce light penetration, and modify benthic community composition – including seagrass and phanerogam density, epibenthic and meiobenthic species composition (Verde and McCloskey 1998; Stoner et al. 2011; Stoner, Yeager, and Layman 2014; Stoner, Yeager, Sweatman, et al. 2014; Zarnoch et al. 2020). Conversely, *Cassiopea* spp. may also bring ecological benefits. Its symbiosis with photosynthetic dinoflagellates suggests a capacity to locally enhance oxygen production and primary productivity (Jantzen et al. 2010; Niggl and Wild 2010; Klein et al. 2017; Durieux et al. 2021). The species can also promote nitrogen recycling while limiting denitrification, a mechanism that may significantly affect nutrient dynamics in coastal environments (Zarnoch et al. 2020). The medusa stage can also support biodiversity by serving as habitat and refuge for marine fauna, and even appears to deter grazers, offering a protective benefit to specific organisms (Niggl and Wild 2010). Furthermore, recent studies highlighted *C. andromeda* jellyfish's biotechnological potential, with promising applications in the nutraceutical and pharmaceutical industries due to its antioxidant‐rich bioactive compounds (De Rinaldis et al. 2021; De Domenico et al. 2023, 2025). Thus, the potential impacts could be site‐specific according to the features of the environment occupied by the species.

Contrary to Banha et al. (2020), this study identifies *C. andromeda* as a valuable model for investigating invasion patterns of thermophilic species under IPCC global warming projections. In future scenarios, temperature could regulate the pace of development and metabolism, influence competition for space, and act as a bottleneck in the Mediterranean Sea. By compiling and discussing the diverse potential ecological implications, this study aims to contribute to a more informed management of Mediterranean coastal ecosystems under climate change scenarios. Once conditions are favorable for the species, *C. andromeda* polyps may spread within the Mediterranean through multiple pathways. Hypotheses suggest that maritime traffic may contribute to its spread, either through ballast water or fouling (Galil et al. 1990; Zenetos et al. 2022). Also, the larvae can attach to submerged surfaces and be passively transported via ships and other floating structures. Upon arrival, confined environments such as harbours, marinas, and coastal lagoons offer optimal conditions for polyp settlement and asexual reproduction (Fernández‐Alías et al. 2021). These habitats often act as initial colonization sites for medusae that may later spread into nearby waters. This dynamic underscores the role of the benthic stage in range expansion and underlines the need to incorporate polyp‐specific physiological thresholds into habitat suitability models. However, over 67% of the available literature on *Cassiopea* spp. is laboratory based (López‐Figueroa et al. 2024) and to enhance the accuracy of our predictions and better assess the role of *C. andromeda* in the environment, mechanistic models should be further refined. This can be achieved by incorporating in situ information, real distribution and abundance of polyps in the ecosystem, and the ecophysiological response to multiple environmental variables (e.g., salinity, O_2_ and CO_2_ concentration), genetic, and phenotypic traits. Habitat suitability models can be constructed using various species‐specific traits (e.g., larvae settlement, Fitt and Costley 1998; feeding rate, Pengpeng et al. 2021; strobilation, Hofmann et al. 1978) and serve as a fundamental tool for analysing patterns of biological invasions in the Mediterranean Sea. These models provide valuable guidance for policymakers and managers when developing and applying early‐warning systems, conservation measures, and monitoring strategies such as those developed in the context of the Marine Strategy Framework Directive (MSFD, European Commission 2008).

## 5 Conclusion

The integration of mechanistic and modelling approaches enabled the creation of *C. andromeda* distribution maps under current and future thermal scenarios. Our findings indicate that the species exhibits a broad thermal tolerance, with the polyp stage able to survive under Mediterranean winter temperatures and showing a strong affinity for high temperatures. Under ongoing climate change, rising temperatures could not only facilitate the spread of *C. andromeda* by allowing polyps to thrive across a wider range of environmental conditions but also promote the successful development and survival of medusae. As a result, this could lead to increased reproduction output, higher population densities, and further geographic spread of the species within the basin. The spatial and temporal increase of occurrence of *C. andromeda* could have site‐specific implications according to the ecological features of the area. Therefore, beyond mapping thermal suitability, these findings underscore the importance of implementing targeted early‐warning systems in coastal areas where polyps are most likely to establish (i.e., harbours, lagoons), as an adaptive strategy to anticipate the multifaceted ecological and socio‐economic impacts of *C. andromeda* proliferation under progressive global warming scenarios.

## Author Contributions

**Lara M. Fumarola:** conceptualization, data curation, formal analysis, investigation, methodology, software, validation, visualization, writing – original draft, writing – review and editing. **Valentina Leoni:** conceptualization, formal analysis, supervision, visualization, writing – review and editing. **Guillaume Marchessaux:** methodology, visualization, writing – review and editing. **Gianluca Sarà:** funding acquisition, resources, writing – review and editing. **Stefano Piraino:** conceptualization, funding acquisition, resources, supervision, writing – review and editing. **Mar Bosch‐Belmar:** conceptualization, data curation, formal analysis, funding acquisition, methodology, resources, software, supervision, validation, visualization, writing – review and editing.

## Conflicts of Interest

The authors declare no conflicts of interest.

## Supporting information


**Table S1:** (a) Published literature used to identify *Cassiopea andromeda* ‘in situ’ thermal ranges in the Mediterranean Sea, the Red Sea, the Atlantic and Indo‐Pacific Oceans. Depth (m), year and coordinates (latitude and longitude) where the specimens were found are also indicated. (b) Online databases used to identify the *C. andromeda* thermal ranges in the Mediterranean Sea and Red Sea. Records of the polyp stage are marked with ‘*’.
**Table S2:** Mean oxygen concentration (mg O2 h−1 g−1 DW−1 ± standard error) measured at each experimental temperature (°C) for Respiration Rate (RR), Net Primary Production (NPP) and Gross Primary Production (GPP). (SE = Standard Error).
**Table S3:** Thermal Habitat Suitability (THS, in percentage) of *Cassiopea andromeda* based on the respiration rate, for the different classes (0.0–0.2, 0.2–0.4, 0.4–0.6, 0.6–0.8 and 0.8–1.0) by month, under Current, RCP 4.5 and RCP 8.5 scenarios of climate change, and the difference of suitability (Δ, as percentage) between the RCP 4.5 scenario and current conditions, and RCP 8.5 scenario and current conditions.

## Data Availability

The data that supports the findings of this study is openly available in Zenodo at http://doi.org/10.5281/zenodo.17202726. DNA barcoding identification has been deposited in the GenBank database under number PX401677 (16S rRNA sequence). The code for the thermal performance curve model “rTPC” applied in this study follows the methodology previously described by Padfield and O’Sullivan 2021 and it is available in Zenodo at http://doi.org/10.5281/zenodo.4561703. The modelling approach applied in this study follows the methodology previously described by Bosch‐Belmar et al. (2022), (https://doi.org/10.3389/fmars.2022.810555). All equations and parameter values used in the model are provided in the main text of this manuscript. Mediterranean monthly mean sea surface temperature (SST) raster files were created from NetCDF (.nc) data downloaded from the Copernicus Marine Service Information portal (https://resources.marine.copernicus.eu). Future scenario maps were generated using forecasted monthly SST for the year 2050, based on modelling projections forced by Representative Concentration Pathways (RCP) 4.5 and 8.5 (IPCC 2021; https://www.ipcc‐data.org).
