# Supplementary material for: Global Warming and the Spread of the Introduced Jellyfish Cassiopea andromeda : Thermal Niche and Habitat Suitability in the Mediterranean Sea
Source: Glob Chang Biol. 2025 Oct 16;31(10):e70548. doi: 10.1111/gcb.70548 (PMC12529649; doi:10.1111/gcb.70548)
Supplement: Supplementary file 1 — Table S1: (a) Published literature used to identify Cassiopea andromeda ‘in situ’ thermal ranges in the Mediterranean Sea, the Red Sea, the Atlantic and Indo‐Pacific Oceans. Depth (m), year and coordinates (latitude and longitude) where the specimens were found are also indicated. (b) Online databases used to identify the C. andromeda thermal ranges in the Mediterranean Sea and Red Sea. Records of the polyp stage are marked with ‘*’. Table S2: Mean oxygen concentration (mg O2 h−1 g−1 DW−1 ± standard error) measured at each experimental temperature (°C) for Respiration Rate (RR), Net Primary Production (NPP) and Gross Primary Production (GPP). (SE = Standard Error). Table S3: Thermal Habitat Suitability (THS, in percentage) of Cassiopea andromeda based on the respiration rate, for the different classes (0.0–0.2, 0.2–0.4, 0.4–0.6, 0.6–0.8 and 0.8–1.0) by month, under Current, RCP 4.5 and RCP 8.5 scenarios of climate change, and the difference of suitability (Δ, as percentage) between the RCP 4.5 scenario and current conditions, and RCP 8.5 scenario and current conditions. [file GCB-31-e70548-s001.docx]

**Global Warming and the Spread of the Introduced Jellyfish  *Cassiopea andromeda*: Thermal Niche and Habitat Suitability in the Mediterranean Sea**

Lara M. Fumarola^1,2^, Valentina Leoni^1^, Guillaume Marchessaux^3^, Gianluca Sarà ^4,5^,

Stefano Piraino^1,2,5^, Mar Bosch-Belmar^1,4,5^

^1^ Consorzio Nazionale Interuniversitario per le Scienze del Mare (Co.N.I.S.Ma.), p.le Flaminio 9, 00196 Roma

^2^ Dipartimento di Scienze e Tecnologie Biologiche ed Ambientali (Di.S.Te.B.A), University of Salento, via per Monteroni, 73100 Lecce, Italy

^3^Aix Marseille Univ, Université de Toulon, CNRS, IRD, MIO, Marseille, France

^4^ Laboratory of Ecology, Department of Earth and Marine Science (DiSTeM), University of Palermo, Palermo, Italy

^5^National Biodiversity Future Center, Palermo (NBFC), 90133, University of Palermo, Italy

*Corresponding author: [laramarastella.fumarola@unisalento.it](mailto:laramarastella.fumarola@unisalento.it)

Doi:10.1111/gcb.70548

**Supplementary material**

**Table S1**. **a)** Published literature used to identify *Cassiopea andromeda* ‘*in situ*’ thermal ranges in the Mediterranean Sea, the Red Sea, the Atlantic and Indo-Pacific Oceans. Depth (m), year and coordinates (latitude and longitude) where the specimens were found is also indicated. **b)** Online databases used to identify the *C. andromeda* thermal ranges in the Mediterranean Sea and Red Sea. Records of the polyp stage are marked with ‘*’.

a.

| **Reference** | **Location** | **Country** | **Depth (m)** | **Year** | **Latitude** | **Longitude** | **Temperature (°C)** |
| --- | --- | --- | --- | --- | --- | --- | --- |
| **Mediterranean Sea records** | | | | | | | |
| Schafer, 1955 | Neokameni | Greece | NA | 1955 | 36.39725 | 25.39874 | 36.0 |
| Goy *et al.,* 1988 | Jounieh | Lebanon | NA | 1988 | 33.98505 | 35.62715 | NA |
| Spainer *et al.,* 1989 | NA | Israel | NA | 1988 | 32.83333 | 34.99083 | NA |
| Galil *et al.,* 1990 | Neve Yam | Israel | 0–5.0 | 1988 | 32.67919 | 34.92741 | NA |
| Çevik *et al.,* 2006 | Iskenderun Bay | Turkey | 0.7 | 2005 | 36.112 | 36.431 | 34.0 |
| Özgür *et al.,* 2008 | Ölüdeniz Lagoon, | Turkey | 0–18.0 | 2003–2004 | 36.55 | 29.1 | 17.0–30.0 |
| Schembri *et al.,* 2010 | Marsamxett Harbour | Malta | 3.5–6.0 | 2009 | 35.53546 | 4.302113 | 14.0 |
| Çardak *et al.,* 2011 | Gulf of Antalya | Turkey | 25.0 | 2009 | 36.46 | 30.35 | 22.9 |
| Katsanevakis *et al.,* 2011 | Paros Island | Greece | 0–10.0 | 2011 | 37.092 | 25.149 | 24.5 |
| Zenetos *et al.,* 2011 | Paros Island | Greece | 0–10.0 | 2010 | 37.085644 | 25.14883 | 22.6 |
| Zenetos *et al.,* 2011 | S. Evvoikos | Greece | NA | 2010 | 38.83171 | 24.55928 | 24.3 |
| Gülşahin & Tarkan, 2012 | Inbükü | Turkey | 4.0–9.0 | 2012 | 36.4729 | 28.044 | 23.0 |
| Kalogirou, 2012 | Rhodes Island | Greece | 0–1.0 | 2011 | 36.18865 | 27.76731 | 27.0 |
| Lakkis *et al*., 2013 | Jounieh | Lebanon | NA | 1987 | 33.98505 | 35.62715 | NA |
| Siokou *et al.,* 2013 | Lattakia | Syria | NA | 2012 | 35.54427 | 35.75808 | 21.0 |
| Ounifi- Ben Amor *et al*., 2015 | Haouaria | Tunisia | 0–2.0 | 2013 | 37.05752 | 10.99299 | 19.0 |
| Ounifi- Ben Amor et al., 2015 | Haouaria | Tunisia | 0–2.0 | 2013 | 37.27761 | 9.887559 | 20.0 |
| Özbek & Öztürk, 2015 | Asin Bay | Turkey | 0.5 | 2014 | 37.27487 | 27.61048 | 21.0 |
| Cillari *et al.,* 2018 | Palermo | Italy | 0–7.0 | 2014 | 38.0722 | 13.2209 | 16.0 |
| Deidun *et al*., 2018 | Salini Nature Reserve | Malta | 0.5–1.0 | 2014 | 35.9491 | 14.4241 | 13.3–14.5 |
| Yokes *et al.,* 2018 | Itea | Greece | 0–4.0 | 2018 | 38.43803 | 22.42044 | 27.2 |
| Crocetta *et al.,* 2021 | Janzour | Libya | 4.0 | 2021 | 32.8394 | 12.9532 | 16.0 |
| De Rinaldis *et al.,* 2021 | Augusta | Italy | NA | 2014 | 37.24149 | 15.23455 | 28.0 |
| Mammone et al., 2023 | Palermo | Italy | 0–7.0 | 2017–2018 | 38.0722 | 13.2209 | 14.1–26.6 |
| Fernandez-Alias, 2024 | Mar Menor | Spain | NA | 2017 | 37.78445 | -0.80048 | 27.0 |
| Marambio et al., 2025 | Marina Aguadulce | Spain | 5.0 | 2023 | 36.8085 | -2.557 | 14.2–14.5 |
| Ramos-Pérez et al., 2025 | Mar Menor | Spain | 0.5–2.0 | 2023 | 37.78445 | -0.80048 | 21.4–31.5 |
| **Red Sea records** | | | | | | | |
| Fricke, 1996 | Sharm El Sheik | Egypt | 95.0 | 1996 | NA | NA | NA |
| Holland *et al.,* 2004 | El Ghardaqa | Egypt | 0–10.0 | 2003 | 33.515 | 27.66 | 29.0 |
| Niggl & Wild, 2010 | Gulf of Aqaba | Jordan | 0–20.0 | 2008 | 34.996 | 29.526 | 27.0 |
| Aljbour *et al.,* 2018 | Gulf of Aqaba | Jordan | 14.0–19.0 | 2016 | 34.95 | 29.526 | 27.0 |
| **Atlantic Ocean records** | | | | | | | |
| Holland *et al.,* 2004 | Florida Key | USA | 0–10.0 | 2003 | 81.122 | 25.146 | 31.0 |
| Holland *et al.,* 2004 | Bermuda | UK | 0–10.0 | 2003 | 64.785 | 32.287 | 30.0 |
| Holland *et al.,* 2004 | Bermuda | UK | 0–10.0 | 2003 | 64.863 | 32.263 | 28.0 |
| Morandini *et al.,* 2017* | Cabo Frio | Brazil | 0–10.0 | 2008–2012 | -22.8785 | -42.021 | 26.6–28.7 |
| Thè *et al.,* 2020 | Acaraú | Brazil | 0–5.0 | 2018 | -2.83535 | -40.1441 | 27.8 |
| Thè *et al.,* 2020 | Acaraú | Brazil | 0–5.0 | 2018 | -2.83535 | -40.1441 | 31.5 |
| Thè *et al.,* 2020 | Acaraú | Brazil | 0–5.0 | 2019 | -2.83535 | -40.1441 | 31.1 |
| Thè *et al.,* 2020 | Acaraú | Brazil | 0–5.0 | 2019 | -2.83535 | -40.1441 | 28.6 |
| Thè *et al.,* 2020 | Itarema | Brazil | 0–5.0 | 2018 | -2.91485 | -39.9073 | 31.7 |
| Thè *et al.,* 2020 | Itarema | Brazil | 0–5.0 | 2018 | -2.91485 | -39.9073 | 31.8 |
| Thè *et al.,* 2020 | Itarema | Brazil | 0–5.0 | 2019 | -2.91485 | -39.9073 | 34.0 |
| Thè *et al.,* 2020 | Itarema | Brazil | 0–5.0 | 2019 | -2.91485 | -39.9073 | 30.4 |
| Muffett & Miglietta, 2023 | Florida Key | USA | 0–2.0 | 2021 | 24.69396 | 81.098051 | 29.1–35.7 |
| Gueroun *et al.,* 2024 | Tenerife | Spain | 0–5.0 | 2023 | 28.498002 | -16.202625 | 19.0–26.0 |
| **Indo-Pacific Ocean records** | | | | | | | |
| Holland *et al.,* 2004 | Honolulu | USA | 0–10.0 | 2003 | 157.877 | 21.297 | 25.0 |
| Prasade *et al.,* 2016* | Gulf of Kutch | India | 0–5.0 | 2013 | 22.434 | 69.045 | 20.4–21.8 |
| Karunarathne *et al.,* 2020 | Jaffna | Sri Lanka | 0–3.0 | 2017 | 80.017 | 9.595 | 25.7 |
| Karunarathne *et al.,* 2020 | Jaffna | Sri Lanka | 0–3.0 | 2018 | 81.245 | 8.506 | 34.7 |
| Kumawat *et al.,* 2022 | Okha | India | 0–5.0 | 2017–2020 | 22.271831 | 69.210111 | 21.0–30.0 |
| Aravind, 2024 | Al Marjan Island | United Arabic Emirates | 0–5.0 | 2021 | 25.669847 | 55.7405 | 20.8–34.2 |

NA: Not available

b.

| **Online database** | | | | | | **Link** | | | |
| --- | --- | --- | --- | --- | --- | --- | --- | --- | --- |
| Cassiopea Base | | | | | | [www.cassiopeabase.org](http://www.cassiopeabase.org/) | | | |
| Jellyfish Dataset Initiative (JeDI) | | | | | | www.bco-dmo.org | | | |
| Ocean Biodiversity of Information System (OBIS) | | | | | | www.obis.org | | | |
| Global Biodiversity Information Facility (GBIF) | | | | | | www.gbif.org | | | |
| MedusApp | | | | | | [www.medusapp.net](http://www.medusapp.net/) | | | |
| JellyWatch | | | | | | [www.jellywatch.org](http://www.jellywatch.org/) | | | |
| Observadores del Mar | | | | | | [www.observadoresdelmar.es](http://www.observadoresdelmar.es/) | | | |
| The Mediterranean Science Commission (CIESM) | | | | | | www.ciesm.org | | | |
| The Policy Oriented Marine Environmental Research in the Southern European Seas (PERSEUS) | | | | | | www.perseus-net.eu | | | |
| Copernicus Ocean Products | | | | | | www.data.marine.copernicus.eu/products | | | |
| **Online database** | **Area** | **Location** | **Country** | **Depth (m)** | **Year** | | **Latitude** | **Longitude** | **Temperature (°C)** |
| Observadores del Mar | Mediterranean Sea | Cala Aiguafreda | Spain | NA | 2023 | | 41.96435 | 3.229169 | 13.4 |
| Observadores del Mar | Mediterranean Sea | El Masnou | Spain | NA | 2023 | | 41.47497 | 2.307545 | 13.4 |
| Medusapp | Mediterranean Sea | Isla Plana | Spain | NA | 2023 | | 37.57146 | -1.20804 | 28.3 |
| Copernicus Ocean products | Red Sea | Gulf of Aqaba | Jordan | NA | 2020–2023 | | 34.996 | 29.526 | 27.–34 |

**Table S2**. Mean oxygen concentration (mg O_2_ h^-1^ g^-1^ DW^-1^ ± standard error) measured at each experimental temperature (°C) for Respiration Rate (RR), Net Primary Production (NPP) and Gross Primary Production (GPP). (SE = Standard Error)

| **Temperature (°C)** | **RR** (**mgO_2_ h^-1^g^-1^DW^-1^) ±SE** | **NPP (mgO_2_ h^-1^g^-1^DW^-1^) ±SE** | **GPP (mgO_2_ h^-1^g^-1^DW^-1^) ±SE** |
| --- | --- | --- | --- |
| **12** | 0.08±0.02 | 0.09±0.02 | 0.17±0.03 |
| **14** | 0.17±0.02 | 0.17±0.03 | 0.34±0.03 |
| **16** | 0.08±0.02 | 0.15±0.04 | 0.23±0.05 |
| **18** | 0.11±0.02 | 0.14±0.03 | 0.25±0.05 |
| **20** | 0.24±0.06 | 0.22±0.03 | 0.46±0.07 |
| **22** | 0.18±0.04 | 0.19±0.02 | 0.37±0.06 |
| **24** | 0.32±0.05 | 0.37±0.04 | 0.69±0.08 |
| **26** | 0.27±0.05 | 0.34±0.04 | 0.61±0.09 |
| **28** | 0.31±0.03 | 0.43±0.05 | 0.75±0.09 |
| **30** | 0.33±0.05 | 0.36±0.05 | 0.68±0.09 |
| **32** | 0.38±0.02 | 0.39±0.03 | 0.78±0.04 |
| **34** | 0.61±0.07 | 0.50±0.03 | 1.12±0.09 |
| **36** | 0.58±0.07 | 0.42±0.07 | 1.01±0.08 |
| **38** | 0.22±0.08 | 0.20±0.08 | 0.42±0.15 |
| **40** | 0 (died) | 0 (died) | 0 (died) |

**Table S3.** Thermal Habitat Suitability (THS, in percentage) of *Cassiopea andromeda* based on the respiration rate, for the different classes (0.0–0.2, 0.2–0.4, 0.4–0.6, 0.6–0.8 and 0.8–1.0) by month, under Current, RCP 4.5 and RCP 8.5 scenarios of climate change, and the difference of suitability (Δ, as percentage) between the RCP 4.5 scenario and current conditions, and RCP 8.5 scenario and current conditions.

| **Class** | **Month** | **Scenarios** | | | **Predicted shifts in THS** | |
| --- | --- | --- | --- | --- | --- | --- |
|  |  | **Current** | **RCP 4.5** | **RCP 8.5** | **Δ (RCP 4.5 - Current)** | **Δ (RCP 8.5-Current)** |
| 0.0 - 0.2 | January | 43.57 | 12.15 | 11.14 | -31.42 | -32.43 |
| 0.2 - 0.4 | January | 55.24 | 86.65 | 87.66 | 31.41 | 32.42 |
| 0.4 - 0.6 | January | 0.40 | 0.41 | 0.41 | 0.01 | 0.01 |
| 0.6 - 0.8 | January | 0.40 | 0.41 | 0.41 | 0.01 | 0.01 |
| 0.8 – 1.0 | January | 0.39 | 0.39 | 0.39 | 0 | 0 |
|  | | | | | | |
| 0.0 - 0.2 | February | 66.89 | 18.33 | 14.77 | -48.56 | -52.12 |
| 0.2 - 0.4 | February | 31.92 | 80.47 | 84.03 | 48.55 | 52.11 |
| 0.4 - 0.6 | February | 0.40 | 0.40 | 0.40 | 0 | 0 |
| 0.6 - 0.8 | February | 0.40 | 0.40 | 0.40 | 0 | 0 |
| 0.8 – 1.0 | February | 0.39 | 0.39 | 0.39 | 0 | 0 |
|  | | | | | | |
| 0.0 - 0.2 | March | 67.84 | 12.96 | 12.62 | -54.88 | -55.22 |
| 0.2 - 0.4 | March | 30.98 | 85.84 | 86.18 | 54.86 | 55.20 |
| 0.4 - 0.6 | March | 0.40 | 0.40 | 0.40 | 0 | 0 |
| 0.6 - 0.8 | March | 0.40 | 0.40 | 0.40 | 0 | 0 |
| 0.8 – 1.0 | March | 0.39 | 0.39 | 0.39 | 0 | 0 |
|  | | | | | | |
| 0.0 - 0.2 | April | 45.18 | 1.94 | 6.11 | -43.24 | -39.07 |
| 0.2 - 0.4 | April | 53.63 | 96.87 | 92.69 | 43.24 | 39.06 |
| 0.4 - 0.6 | April | 0.40 | 0.41 | 0.41 | 0.01 | 0.01 |
| 0.6 - 0.8 | April | 0.40 | 0.41 | 0.41 | 0.01 | 0.01 |
| 0.8 – 1.0 | April | 0.38 | 0.38 | 0.38 | 0 | 0 |
|  | | | | | | |
| 0.0 - 0.2 | May | 0.43 | 0.40 | 0.40 | -0.03 | -0.03 |
| 0.2 - 0.4 | May | 97.82 | 94.34 | 90.78 | -3.48 | -7.04 |
| 0.4 - 0.6 | May | 0.98 | 4.47 | 8.03 | 3.49 | 7.05 |
| 0.6 - 0.8 | May | 0.40 | 0.40 | 0.40 | 0 | 0 |
| 0.8 – 1.0 | May | 0.38 | 0.38 | 0.38 | 0 | 0 |
|  | | | | | | |
| 0.0 - 0.2 | June | 0.40 | 0.40 | 0.40 | 0 | 0 |
| 0.2 - 0.4 | June | 18.73 | 22.14 | 9.54 | 3.41 | -9.19 |
| 0.4 - 0.6 | June | 79.10 | 75.73 | 85.26 | -3.37 | 6.16 |
| 0.6 - 0.8 | June | 1.39 | 1.34 | 4.41 | -0.05 | 3.02 |
| 0.8 – 1.0 | June | 0.38 | 0.38 | 0.38 | 0 | 0 |
|  | | | | | | |
| 0.0 - 0.2 | July | 0.40 | 0.41 | 0.41 | 0.01 | 0.01 |
| 0.2 - 0.4 | July | 2.60 | 3.03 | 2.66 | 0.43 | 0.06 |
| 0.4 - 0.6 | July | 29.95 | 36.72 | 49.28 | 6.77 | 19.33 |
| 0.6 - 0.8 | July | 64.79 | 59.36 | 45.12 | -5.43 | -19.67 |
| 0.8 – 1.0 | July | 2.25 | 0.48 | 2.54 | -1.77 | 0.29 |
|  | | | | | | |
| 0.0 - 0.2 | August | 0.38 | 0.38 | 0.41 | 0 | 0.03 |
| 0.2 - 0.4 | August | 0.81 | 1.08 | 1.01 | 0.27 | 0.20 |
| 0.4 - 0.6 | August | 28.91 | 13.85 | 16.59 | -15.06 | -12.32 |
| 0.6 - 0.8 | August | 61.01 | 61.52 | 67.68 | 0.51 | 6.67 |
| 0.8 – 1.0 | August | 8.89 | 23.17 | 14.32 | 14.28 | 5.43 |
|  | | | | | | |
| 0 - 0.2 | September | 0.40 | 0.41 | 0.41 | 0.01 | 0.01 |
| 0.2 - 0.4 | September | 2.85 | 5.84 | 1.90 | 2.99 | -0.95 |
| 0.4 - 0.6 | September | 57.49 | 60.19 | 34.43 | 2.70 | -23.06 |
| 0.6 - 0.8 | September | 36.09 | 31.73 | 56.84 | -4.36 | 20.75 |
| 0.8 – 1.0 | September | 3.17 | 1.83 | 6.43 | -1.34 | 3.26 |
|  | | | | | | |
| 0.0 - 0.2 | October | 0.40 | 0.40 | 0.40 | 0 | 0 |
| 0.2 - 0.4 | October | 33.51 | 22.39 | 11.14 | -11.12 | -22.37 |
| 0.4 - 0.6 | October | 54.06 | 70.77 | 69.38 | 16.71 | 15.32 |
| 0.6 - 0.8 | October | 11.64 | 6.05 | 18.65 | -5.59 | 7.01 |
| 0.8 – 1.0 | October | 0.39 | 0.39 | 0.43 | 0 | 0.04 |
|  | | | | | | |
| 0.0 - 0.2 | November | 0.40 | 0.40 | 0.40 | 0 | 0 |
| 0.2 - 0.4 | November | 69.93 | 64.63 | 54.26 | -5.30 | -15.67 |
| 0.4 - 0.6 | November | 28.88 | 34.18 | 44.55 | 5.30 | 15.67 |
| 0.6 - 0.8 | November | 0.40 | 0.40 | 0.40 | 0 | 0 |
| 0.8 – 1.0 | November | 0.39 | 0.39 | 0.39 | 0 | 0 |
|  | | | | | | |
| 0.0 - 0.2 | December | 11.71 | 2.97 | 0.86 | -8.74 | -10.85 |
| 0.2 - 0.4 | December | 85.52 | 95.83 | 93.33 | 10.31 | 7.81 |
| 0.4 - 0.6 | December | 1.98 | 0.40 | 5.02 | -1.58 | 3.04 |
| 0.6 - 0.8 | December | 0.40 | 0.40 | 0.40 | 0 | 0 |
| 0.8 – 1.0 | December | 0.39 | 0.39 | 0.39 | 0 | 0 |

**References**

Aravind, A. 2024. *Reproductive Biology of the Jellyfish Outbreak Along the UAE Coastal Waters*. United Arab Emirates University, College of Science PhD dissertation thesis.

Çardak, M., E. Özgür Özbek, and T. Kebapçıoğlu. 2011. “The New Location Record of *Cassiopea andromeda* (Forsskål, 1775) From the Gulf of Antalya, Levantine Coast of Turkey, Eastern Mediterranean.” In *First National Workshop on Jellyfish and Other Gelatinous Species in Turkish Marine Waters. Turkish Marine Research Foundation*, vol. 35.

Crocetta, F., S. Al Mabruk, E. Azzurro, et al. 2021. “New Alien Mediterranean Biodiversity Records (November 2021).” *Mediterranean Marine Science* 22, no. 3: 724–746. <https://doi.org/10.12681/mms.26668> .

Fernández‐Alías, A. 2024. “Ecología de los escifozoos. Universidad de Murcia.” PhD dissertation thesis.

Fricke, H. 1996. “On the Pathways of the ‘Pola’ Expeditions. Deep‐Water Exploration of the Red Sea by Submersible. In: Deep Sea and Extreme Shallow‐Water Habitats: Affinities and Adaptations.” *Biosystematics and Ecology* 11: 67–89.

Goy, J., S. Lakkis, and R. Zeidane. 1988. “Les Meduses de la Méditerranée Orientale. Rapport de la Commission International Pour l'Exploration Scientifique de la Mer.” *Méditerranée* 31, no. 2: 299 CIESM: Monaco.

Gülşahin, N., and A. N. Tarkan. 2012. “Occurrence of the Alien Jellyfish *Cassiopea andromeda* (Scyphozoa: Rhizostomeae: Cassiopeidae) in Hisarönü Bay, Muğla.” *Turkey. Biharean Biologist* 6, no. 2: 132–133.

Kalogirou, S. 2012. “Occurrence of the Non‐Indigenous Scyphomedusa *Cassiopea andromeda* (Forsskål, 1775) in Rhodes Island (SE Aegean Sea). In New Mediterranean Biodiversity Records.” *Mediterranean Marine Science* 13, no. 1: 162. . <https://doi.org/10.12681/mms.13771>.

Katsanevakis, S. 2011. “Rapid Assessment of the Marine Alien Megabiota in the Shallow Coastal Waters of the Greek Islands, Paros and Antiparos, Aegean Sea.” *Aquatic Invasions* 6, no. 1: 133–137. <https://doi.org/10.3391/ai.2011.6.S1.030>.

Kumawat, T., R. Saravanan, K. Vinod, et al. 2022. “Scyphozoan Jellyfish Diversity and Distribution Along the North‐Eastern Arabian Sea, Off Gujarat Coast, India.” *Indian Journal Of Fisheries* 69, no. 3: 126–134. <https://doi.org/10.21077/ijf.2022.69.3.110008-15>.

Lakkis, S. 2013. “Le Zooplancton Des Eaux Marines Libanaises (Méditerranée Orientale): Biodiversité, Biologie, Biogéographie.” *ARACNE éditrice Srl* 14: 76334.

Mammone, M., M. Bosch‐Belmar, G. Milisenda, et al. 2023. “Reproductive Cycle and Gonadal Output of the Lessepsian Jellyfish *Cassiopea andromeda* in NW Sicily (Central Mediterranean Sea).” *PLoS One* 18, no. 2: e0281787. <https://doi.org/10.1371/journal.pone.0281787>.

Marambio, M., M. Pascual‐Torner, U. Tilves,  et al. 2025. The Westernmost Record of the Scyphomedusa *Cassiopea andromeda* (Forskål, 1775) in the Mediterranean: Marine Citizen Science Contributions to Invasive Species Detection and Monitoring. *Environmental Management,*  (<https://doi.org/10.1007/s00267-025-02289-w> .

Ounifi‐ Ben Amor, K., Μ. Rifi, R. Ghanem, I. Draeif, J. Zaouali, and J. Ben Souissi. 2015. “Update of Alien Fauna and New Records From Tunisian Marine Waters.” *Mediterranean Marine Science* 17, no. 1: 124–143. <https://doi.org/10.12681/mms.1371>.

Özbek, E. Ö., and B. Öztürk. 2015. “The New Location Record of *Cassiopea andromeda* (Forsskål, 1775) From Asin Bay, Gulf of Güllük, Muğla, Aegean Coast of Turkey.” *Journal of Black Sea/Mediterranean Environment* 21, no. 1: 96–101.

Schafer, W. 1955. “Eine Qualle Aus Dem Indischen Ozean in Der Agais.” *Natur Volk* 85: 241–245.

Siokou, I., A. Ates, D. Ayas, et al. 2013. “New Mediterranean Marine Biodiversity Records (June 2013).” *Mediterranean Marine Science* 14, no. 1: 238–249. <https://doi.org/10.12681/mms.915>.

Spanier, E. 1989. “Swarming of Jellyfishes Along the Mediterranean Coast of Israel.” *Israel Journal of Zoology* 36: 55–56.

Yokeş, M., V. Andreou, R. Bakiu, et al. 2018. “New Mediterranean Biodiversity Records (November 2018).” *Mediterranean Marine Science* 19: 3. <https://doi.org/10.12681/mms.19386>.

Zenetos, A., S. Katsanevakis, D. Poursanidis, et al. 2011. “Marine Alien Species in Greek Seas: Additions and Amendments by 2010. Mediterranean Marine.” *Science* 12, 1: 95–120. <https://doi.org/10.12681/mms.55>.
